# ‘All Avatars Aren't We’: Football and the experience of football-themed digital content during a global pandemic

**DOI:** 10.1177/10126902211021529

**Published:** 2022-06

**Authors:** Garry Crawford, Alex Fenton, Simon Chadwick, Stefan Lawrence

**Affiliations:** 7046 University of Salford, UK; 7046 University of Salford, UK; 55493 EM LYON Business School, France; 1713 Newman University, UK

**Keywords:** consumption, COVID-19, esports, experience, football, fandom, pandemic, participation, theming

## Abstract

This paper explores the contemporary nature of association football consumption. In particular, we argue that the coronavirus 2019 pandemic reveals the contemporary and particular nature of the relationship between football and its supporters, which is increasingly focused on the consumption of themed digital participatory experiences. During this pandemic, what fans missed was not only live football, but also the sporting ‘experience’ and the opportunities for participation that this provides. Hence, here we saw fans, clubs and media providers employing new digital technologies to create themed experiences that facilitated (and mediated) participation and interaction. Following Žižek (2014), we suggest that the coronavirus 2019 pandemic can be understood as a global mega event that creates a seismic, reality alerting schism, whose aftermath requires new ontologies and theories. Our response is to utilise a number of key and illustrative examples and to offer a new synthesis of theories and literatures, most notably, on the experience society, theming, participatory culture, neoliberalism and digital culture. This new context and (re)combination of theories then provides a new, and essential, perspective that reveals a great deal about the contemporary nature of the sport, what fans buy into, and also, how this may change post pandemic.

## Introduction

This paper explores contemporary association football (referred to as ‘football’, here on) fandom, relating this discussion to changing patterns of digital consumption. Moreover, we examine how the recent coronavirus disease 2019 (COVID-19) global pandemic has revealed the contemporary and particular nature of the relationship between football and its supporters, which is increasingly focused on the consumption of themed digital participatory experiences.

A key tenet of this paper is that during the pandemic, what fans missed was not only live football, but also the sporting experience and the opportunities for participation that this provides. Hence, during the pandemic, we saw fans, clubs, and media providers working separately and together to use digital technologies to create themed experiences that facilitated continued (and mediated) participation and interaction. This then reveals a great deal about the contemporary nature of the sport, what fans buy into, and also, how this may change post-pandemic.

The paper begins with a brief discussion of the impact of COVID-19 upon professional football, specifically in the UK – and the examples used here, and throughout the paper, have been purposefully selected to illustrate particular characteristics of digitally mediated football during the pandemic.

The next section provides a brief overview of what we see as the key trends in the academic literature on football fandom and contends that this paper contributes to, and advances, a new and developing ‘digital football studies’ – the literature that focuses on the increasingly central role of digital media in the production and consumption of sport.

Next, we chart the changing nature of consumer society. Here, we argue that due to a number of inherent weaknesses within consumer society we have seen an evolution of a new kind of economy, based around the creation and consumption of experiences.

Thereafter, the paper identifies that, although at the turn of the 20th-century television seemed to be the dominant way most fans consumed football, for a newer generation of supporters, new digital technologies, such as social media and mobile devices, offer both greater convenience and more opportunities for participation. However, importantly, we argue that this does not necessarily provide fans with any greater access, power, or connection, but in many ways, may further isolate and disempower supporters.

The following section highlights the central role of theming in an experience society. Theming is not necessarily new in the sale and consumption of sport; however, we suggest that new digital technologies offer greater opportunities for the creation and consumption of participatory football-themed experiences.

We then reflect more broadly on the development of an experience society and participatory culture and consider if this can be understood (more positively) as changes in audience needs and demands, or (more negatively) as part of a wider neoliberal culture and economy. Then, finally, the paper concludes by asking if the COVID-19 pandemic may have accelerated this shift to a primary engagement with football through new digital technologies.

Therefore, although undoubtedly there is an existing (and developing) literature on areas such as sport and the digital, theming in sport, and sport in an experience society, none have brought these (and other) strands together as we do here, in a way that allows for a better understanding of the contemporary consumption of football – as a themed digital participatory experience. The synthesis of these, and various other, literatures in this paper not only advances our understanding of the contemporary consumption of football, but moreover, is crucial for considering the increased shift towards the consumption of digital experiences we witnessed during the COVID-19 pandemic, and we would suggest, will continue to accelerate post-pandemic. This context and (re)combination of theories then provides a new, and essential, perspective that reveals a great deal about the contemporary nature of the sport, what fans buy into, and also, how this may change post-pandemic.

## COVID-19 and football in a time of a global pandemic

On 12 March 2020, the English and Scottish Premier Leagues and Football Associations, Scottish Professional Football League, English Football League, and Women's Super League collectively suspended all professional football matches – the first time football had paused in this way since World War Two. This similarly occurred, at around the same time, in most other countries around the world. The last football league to hold out against closure was the Belarusian Premier League, which in March 2020 signed at least 10 new television deals, (*The Guardian, 2020a*). However, live matches in this league, like all others, soon ceased.

This unprecedented pausing of live sporting experiences created major logistical and financial problems (Stone, 2020a), as it reduced the flow of content of major sporting brands. Once the supply of traditional content was halted, football clubs and media providers were left with a major issue of how to keep fans entertained and engaged.

Response was that many football fans and clubs turned to digital media as a means to fill the void left by live sport. One of the first to do so was Leeds United, whose game against Cardiff City on 15 March 2020 had been postponed indefinitely, who decided to let the video game Federation Internationale de Football Association (*FIFA 20*) simulate the game, broadcasting this live on social media (Sobot, 2020).

The game was promoted on the day on the Leeds United official Twitter feed with one simple tweet, ‘Anyone else bored? How about we let #FIFA20 decide today's result’. The game ended in a 3-3 draw and attracted over 50,000 views on Periscope (Sobot, 2020). The response from those who followed the game on social media was mixed. Many fans were clearly engaged and commented on the game as if they were following a ‘real’ game (a physical, in-stadium, football match), such as celebrating the goals. Some seemed largely bemused by it all, but most seemed to be, at least in part, entertained, and were actively joined in by tweeting comments during the game – such as one Leeds fan tweeted, ‘all avatars aren’t we’ with a laughing emoji, a play on the famous Leeds’ chant of ‘all Leeds aren’t we’.

Another early example was on 4 April 2020, the day that Wolverhampton Wanderers (Wolves) had been due to play their local rivals Aston Villa in a Premier League game. The (then) Wolves player Diogo Jota took on Villa's Ezri Konsa at *FIFA 20*, playing for their respective teams, in a game that was streamed live on Twitch. The contest had live commentary from Wolves TV commentators, match highlights were posted on Twitter, and local newspapers, like Wolverhampton's *Express and Star*, ran articles on the match much as they would have for any Premier League game. Another alternative was presented by the non-league outfit, Whitehawk in their game against Guernsey. Whitehawk and Guernsey decided at the end of March 2020 that they would devise a script for their scheduled game, and then post on Twitter, as if reporting on a live match – which in the end, turned out to be a 12-goal thriller that finished six goals apiece. However, probably the biggest and most visible organised event was the Ultimate QuaranTeam eSports contest.

The idea of the tournament originally came from the media team at Leyton Orient, who invited 63 other English teams to join them in a *FIFA 20* knockout tournament. However, the teams involved grew far beyond the original list and outside of the UK, with, in the end, 128 teams from 16 different countries participating (Ouzia, 2020). Some of the teams were controlled by players from the respective clubs, like Crystal Palace player Andros Townsend, and Blackburn Rovers’ Bradley Dack. Other clubs enlisted fans to represent them, while some, like the eventual winners’ Wolves, already had professional *FIFA* players signed to represent the club. All of the games were then streamed live on Twitch, and all major gambling companies took bets on them like they would for any other football match. Clubs also posted social media updates in exactly the same way they would with a ‘real’ match.

Fans also sought to produce or play out their own football-related alternatives. Examples included a series of social media chain posts, where individuals posted their favourite football-related pictures, along with copied text about how much they were missing football and then a nominated person whom they were asking to carry on the chain, or similarly, a chain post asking a nominated person(s) to share pictures of their five favourite footballers. Gamers have also been taking to playing football simulations in large numbers. Sports Interactive made the latest version of their video game *Football Manager* available for free at the end of March 2020 for 1 week, but due to popular demand, extended this for another week (Hawkins, 2020). However, even before the game was made free, during the first weeks of the first coronavirus lockdown in the UK, the game was racking up record numbers of players on the video game service Steam (Gallagher, 2020).

These are, of course, just some illustrative examples, as media outlets, football clubs (as well as other sports), and fans, sought to create and deliver content to fill the void created by the absence of live matches. However, what unites all of these (and numerous other) examples is that they all provide fans with not only an alternative (most often digital) *themed experience*, but also an opportunity to *participate* in this, such as by playing out sports-themed video games, or posting or commenting on social media.

Undoubtedly, therefore, the consumption of football, like much of the rest of society, changed radically during the COVID-19 pandemic. Hence, we would suggest, that this pandemic constitutes what Žižek (2014) would see as a global mega-event, similar to the global financial crisis of 2007, which creates a seismic, reality-alerting schism, whose aftermath requires new ontologies and theories – or here we would propose a (re)combination of theories – that better capture emerging social and cultural developments.

## Football fandom and consumption

Consumption has always been part of football, as have all organised spectator sports, but from the mid-20th century onwards, consumption became ever more central to professional football (Crawford, 2004). Undoubtedly, football fundamentally changed in England after 1961 with the removal of the players’ wage cap that had been in place since 1904. This, for sociologists such as Taylor (1969), marked the start of what he saw as a ‘bourgeoisification’ of the game.

From the early 1990s onwards, football then underwent what Giulianotti (2002) defines as a ‘hypercommodification’. Certainly, in the UK, the last decade of the 20th century saw significant changes to the nature and consumption of football. In 1990, the publication of the Taylor report, after the Hillsborough tragedy of the previous year, recommended and led to all professional football stadia in the UK becoming all-seater venues. This was also the year when English football teams were readmitted to European club competition for the first time after a 5-year ban, the men’s English national squad came agonisingly close to cup glory in the 1990 World Cup Finals, losing in the semi-finals to West Germany on penalties. All of this was then followed by the creation of the English FA Premier League, and its subsequent deal with *BSkyB* television (Lawrence and Crawford, 2019).

It is also in this period that we saw the development of a body of work focusing on, what was termed, football's ‘new fandom’. Writers, such as Taylor (1995), Redhead (1997) and King (2002), set out to chart the changing nature of football, but more specifically, argued that we were the creation of a new kind of supporter, what King (2002) referred to as ‘new consumers’ – whose primary means of connecting with the sport was through the consumption of large quantities of official club merchandise (such as replica football shirts) and televised matches.

Although the new fandom literature was undoubtedly a key development in the academic study of football, an inherent problem with much of this work, as with much of what had gone before it, was that this tended to, either explicitly or implicitly, romanticise an (largely imagined) era of traditional (primarily White male) working-class ‘real’ fans, as opposed to what the authors saw as a new ‘inauthentic’ (middle-class and family-based) consumer audience. This was an assumption subsequently challenged by several authors, including Crawford (2004), who argued that a more useful theorisation was to understand all sport fandom as an act of consumption, as this avoids value-laden categories of authenticity.

It is then on the foundations of this early literature that at the turn of the century we saw the rapid growth and establishment of football studies as a significant area of academic research. This includes (but is not limited to) the work of authors, such as Poulton (2007), Millward (2012), Dixon (2013), Gibbons (2014), Doidge (2014), and Pope (2017). These, and others, helped significantly move the discussion of football fandom into new areas, and in ways that left behind old typologies based around outdated ideas of authenticity. For example, this new research gave more consideration to previously marginalised topics, such as female fans (for example Pope, 2017), and Black, Asian, and Minority Ethnic supporters (for example Lawrence and Davis, 2019).

However, much of this literature engaged either directly or indirectly with what David et al. (2017) have referred to as the ‘Murdochization of football media’. That is to say, how the changing relationship between football and broadcast media (and more specifically satellite and cable television) from the early 1990s onwards shaped the nature and consumption of sport.

Lawrence and Crawford (2019) suggested that what we have started to see in recent years is a new wave of football fan literature, which they refer to as ‘digital football studies’. This emerging literature considers the ways fans are now engaging with football via digital technologies, including peer-to-peer live streaming (such as David et al. 2017), podcasts (such as Pipini, 2019, Rivers and Ross, 2019), and video games (such as Crawford, 2015), to name but a few. As Miah (2017: 3) argues, we are witnessing ‘a transition [in sport] from an analogue to a digital way of producing and experiencing sport’. This new literature is therefore engaging with, and documents, how new digital media is increasingly central to the production, consumption and experience of football – and in particular, it is to this literature, that this paper adds, and moves forward.

## Consumption and the experience society

In this section, drawing most notably on the work of Miles (2021), we argue that due to a number of inherent weaknesses within consumer society, we have seen an evolution of a new kind of consumer society, based on the creation and consumption of experiences. This, as we shall argue, has particular consequences for the nature of football and how it is consumed.

The work of Miles (2021) is one of our key starting points, as Miles’ study is both the most up-to-date and also most comprehensive attempt to understand the location of sport in an experience society. However, Miles’ work primarily focuses on the importance of broadcast (the ‘Murdochization of football’), rather than digital, media, and consequently, ideas such as theming and participatory culture are not particularly prominent in his work – certainly not in respect to their role in the consumption of sport. Moreover, Miles’ work was written before the COVID-19 pandemic, and it is our argument here, that it is this that makes even more visible the central role of themed digital participatory experiences in the consumption of football. Hence, the work of Miles (as well as others, such as Pine and Gilmore, 2011) on the experience society offers the first, of several, strands we seek to weave together − which includes, most notably theming, participatory culture, neoliberalism, and digital culture − in order to offer new perspectives and insights into the contemporary consumption of football.

Miles (2021) highlights that there are, at least, five inherent limits to consumer culture. First, as highlighted by Adorno and Horkheimer (1979) consumption never fully delivers what it promises. In particular, capitalism (most notably through advertising) sells false hopes and desires that it never fully delivers on, and hence, ‘perpetually cheats its consumers of what it perpetually promises’ (Adorno and Horkheimer, 1979: 145).

Second, though we may draw on consumer products and resources in the performance of our identities, how others perceive this may not necessarily match our own, or intended, self-image. As Miles (2021: 30) argues ‘one individual's idea of good taste is another's “crass consumerism’”. Then, of course, how others see us often impacts on our own self-image. Hence, for example, the football shirt a fan might initially love may soon lose its appeal if they think others (whose opinion they value) are judging them negatively for wearing it. For example, King (2002) highlights how supporters at clubs like Manchester United who wore replica football shirts, may have thought they were demonstrating their loyalty to the club, but by others, they were often dismissed as not ‘real’ fans.

Third, Miles (2021) argues, that not only do consumer goods never fully deliver on their promise, but neither does their novelty last long. For an item to have any prolonged meaning, it needs to be associated with a particular, or certain types, of experience. As he writes ‘they could be the shoes we wear to go dancing…But in order for the self to feel that it belongs…they need to be more than mere shoes. The shoes are merely one element of a more impactful and less transitory experience’ (Miles, 2021: 30). This, is of course, what some of the new fandom literature failed to grasp – that is, that the meanings people attach to objects, such as a football shirt, matter. As Kendall and Osbaldiston (2010) highlighted, a football shirt is much more than just a piece of synthetic material, and for many, can be a sacred item deeply imbedded in personal narratives.

Fourth, as Bauman (1998) highlights, certain consumers are inevitably ‘flawed’. This describes those who aspire to consume, but for various reasons, such as lack of economic capital, cannot, or certainly cannot at the level they aspire to. And, to not consume, (to use Bauman's terminology) is to become a ‘vagabond’ – an outcast from (consumer) society. Again, links here can be made to the new football fandom literature, which argued that many traditional supporters were being priced out of the game.

Fifth, Miles argues that the consumption of objects, our investment in them, and their power in determining our social status, are less important than they once were. Of course, as highlighted above, the links between how we think something will make us appear, and how it actually does, has always been far from straightforward. However, partially due to increased concerns about excessive consumption and its impact on the environment, Miles (2021: 27) argues, that ‘there are signs that consumers are less drawn to objects as a means of self-expression’.

Hence, Miles (2021: 30) argues, consumption is ‘a never-ending cycle of dissatisfaction’. Although consumer products offer us an almost endless supply of products, ‘they are always transient and empty of permanent meaning’. Hence, ultimately the consumption of goods and products has failed us because they never fully delivered on what they promised, or what we wanted them to.

It has therefore been suggested that what we have increasingly seen from the end of the 20th century onwards, is a shift towards what has been termed an ‘experience society’ (Miles, 2021) or ‘experience economy’ (Pine and Gilmore, 2011). This constitutes a new form of consumer culture, based not upon products or services, but instead on the consumption of staged experiences. As Pine and Gilmore (2011: ix) suggested, ‘in a world saturated with largely undifferentiated goods and services the greatest opportunity for value creation resides in staging experiences’. For Pine and Gilmore, this ‘value creation’ is mutually beneficial for both the producer and the consumer – as the consumer gets a more enjoyable, engaging, and prolonged encounter, while the former profits from being able to sell something customers seem to want.

For Pine and Gilmore (2011), the origins of the experience economy can be found in the Disneyland theme park, but it is the subsequent rapid development of new technologies that have enabled a ‘whole new genre of experience, such as video games, online games, motion-based attractions, 3-D movies, virtual worlds, and augmented reality’ (Pine and Gilmore, 2011: 4). In particular, the development of the experience society is closely linked to technological innovations and informational modes of capitalism that have enabled greater consumer interaction and participation.

Therefore, central to an experience society is participation. As Salen and Zimmerman (2004: 314) succinctly write ‘experience is participation’. While Salen and Zimmerman may be overstating their point, as experience is obviously more than just participation – as experiences are most commonly staged for our consumption – this does highlight the centrality of participation to the experience society. There is certainly no experience without the active participation of individuals or groups in the events that are staged. In particular, the experience society constitutes part of an intertwined process of the increased blurring between acts of consumption and production, or what Toffler (1980) referred to as the rise of the ‘prosumer’.

Of course, we use terms like ‘prosumer’ with a great deal of caution, as experiences are often *sold* to consumers on the basis of their participation. For example, this is particularly the case with video games, which are often promoted on the basis of the gamer's ability to mould the game to their own experience. However, the ability of the gamer to personalise their gaming experiences are often greatly overstated (Crawford, 2012). Video games, like all staged experiences, are limited in what they offer – limited by not only what the technologies involved, but also by the intentions and ideologies of the designers behind the staging of the experience. However, it is certainly the case that individuals often *feel* involved with the experiences they participate in, as these provide a (real or imagined) sense of being involved in the creation of the overall experience. This is because, in an experience society, consumers feel increasingly empowered to make choices about what and how they consume, and hence, feel more invested in it – even if that feeling of empowerment may be largely imagined.

## Football in an experience society

Football (as with other sports) has always been an experience. For fans, historically, this experience came first and foremost from *being there*, at the live event. This changed with the advent of televised football. However, for most fans, watching sport on television has always been a secondary and poor replacement to the live experience. Hence, the aim of many media providers has been to deliver a more engaging experience for those consuming sport at a distance. This they have done by adding elements such as, action replays, expert commentary, and multiple camera angles, which are all aimed at making the televised experience less of a substitute, and in some respects, surpassing the live experience.

However, Jenkins (2006) argues, changes in media consumption patterns are not just top down or technologically driven but can also be shaped by changing audience profiles and needs. In particular, it has been suggested that in recent years we have seen a steady decline in viewing figures for live televised sport, but in kind, a marked increase in the number of streaming sports footage, both live and after the event, most often via mobile devices (Evens, 2017).

Generation-Z consumers (those born between the mid-1990s and 2010) are not watching sport in the same way as older generations did (Özkan, 2017). Rather, for this new generation, live streaming plays a much bigger role in how they consume content, and they are much more likely than older football fans to use streaming apps and unauthorised streaming services (Evens, 2017). This is then, what Rowe et al. (2010: 301) referred to as the ‘post-hegemonic broadcast age’, where sport fans are increasingly consuming sport via digital content and devices. As Evens writes:

It is no secret that live television is under pressure, most significantly by the rise of connected viewing practices, which form part of an impending revolution in how screen media is created, circulated and consumed. The migration from one screen to many, upending traditional business models and provoking multi-platform strategies, challenges traditional television viewership (Evens, 2017: 284).

Engaging with sport on a digital device offers important advantages over broadcast television. First, it allows consumers a more individualised experience – they get to see what they want, when they want it, and where they want it. Second, this more easily facilitates engagement with social media, allowing fans to share content and connect with others online. Digital media therefore replicates, or at least offers a surrogate, for the types of participation and interaction that sport fans have always experienced and desired.

Experiences are social and communal, as they are often undertaken with others, most commonly, friends or family members. This is certainly the case for live sporting events, such as a football match. However, this is also increasingly the case for mediated experiences, such as streaming a football match online, which can also facilitate interaction with others, such as via social media. However, it is important to recognise that it is often the communal nature of experiences that are sold to (individual) consumers – it is the experience of community and belonging that consumers are often, at least partially, buying into, and this is as much true for those following sport online as it is for those attending live games.

However, what happens when the central experience you are selling ceases to be available? As it turned out to be the case from mid-2020 onwards when football leagues and competitions around the world stopped playing. This, we would suggest, helps reveal the contemporary nature of football and its relationship with its fans, and in particular, the central role that theming plays in the experience society and in the staging of football.

## Football-themed experiences

Theming is central to the experience of society. Gottdiener (2001) suggests that the origins of theming can be traced back to American fast-food restaurants, where themes are used to create a consistent and enjoyable customer experience. However, it is evident that theming is, and has been, part of the promotion and sale of sports experiences for a considerable time. For example, items such as scarves and replica shirts have all been sold for decades (if not longer), carrying the brand (or theme) of particular clubs.

However, the real power of themes is in their ability to be applied to other related (or sometimes unrelated) products, services, and experiences, such as sports-themed cafés, like Manchester United's Red Cafés. Themes can be applied and used to sell a wide variety of different things, but Crawford (2015) suggests, that with football one of the most successful here has been football-themed video games.

Crawford (2015) argues that one of the reasons sport-themed video games are so successful, and we would extend this argument to suggest, fit so well within an experience society, is that they offer the gamer a sense of control, individualisation of experience, and involvement. Gamers feel like they are controlling the game action and taking it in a direction that is unique to them – (co)creating the experience. This fits well with sport fans, who have often felt an active part of the clubs and sports they support – such as encapsulated in comments like ‘*we* won on Saturday’ (Sandvoss, 2003).

However, football-themed experiences are not just limited to video games. In recent decades, social media and other new digital technologies, such as (official and unofficial) social media pages, and football-themed apps, have allowed (and fostered) greater supporter involvement and interaction and there is a rapidly growing literature on this (that includes, McCarthy et al., 2014, Vale and Fernandes, 2016, Fenton et al., 2020). As we have set out above, interaction and participation are now an accepted and expected part of the experience society, and it is this, at least in part, that sport fans then miss once some of the possibilities for this are taken away, such as happened in mid-2020.

The response of football clubs, leagues, and media providers to the pandemic is then interesting when viewed in light of the location of sport, and more specifically, football, in an experience society. Part of their response, certainly at the beginning of the pandemic, was to show football matches from alternative leagues that were still playing, such as in Belarus and then later Germany, and while there were no games being played at all, re-showing classic games, such as ITV in the UK did by screening Euro ’96 in its entirety (Kershaw, 2020). However (as set out at the beginning of this paper), clubs, leagues, and media channels also provided a wealth of digital football-themed alternative content to keep supporters engaged, such as using video games or social media to play out cancelled games.

However, there were numerous other ways that football clubs, associations, and football-related businesses sought to remain connected with, and offer opportunities for fans to participate in themed experiences. For example, at the end of March 2020, the English FA launched a mobile app aimed at children, called *Superkicks*, that is filled with football-related tasks, such as designing a football team kit and drawing Wembley Stadium (The FA, 2020). In Japan, the J-League introduced an app called *Remote Cheerer* that allowed fans to use their mobile phones to record their cheers, which were then replayed via speakers into football stadiums during live games (Whiting, 2020). Furthermore, the CEO of the technology company Screach, Robert Rawlinson, outlined how some sport-themed pubs were using social media to continue to engage with their customers while the pubs were closed (Stone, 2020b). This, Rawlinson indicates, included pubs posting on social media videos of crowds in their venues, and asking customers to find themselves in the crowd, or hosting online sport-themed quiz nights. As Rawlinson suggests ‘there's plenty of scope for them [sport-themed pubs] to share lockdown content from the wider sporting world to remind customers that the pub is very much part of their sport *experience*’ (Stone, 2020b, emphasis added). Similarly, many football clubs themselves offered these kinds of activities, like Wolves, for example, hosting online club-themed quiz nights. Additionally, there was a plethora of football-themed user-generated content posted on social media. All of which provided further opportunities for fan social interaction and participation.

Therefore, the consumption of football-themed content during a pandemic illustrates how in an experience society, when the traditional focus of a particular experience is missing, content providers and audiences alike work together to create new and themed experiences. This then allows traditional content providers (such as football clubs) to keep their audience actively engaged. This also provides audiences (in this case, football fans) with the kinds of themed content and participatory experiences that they have become accustomed to.

A final question though, is to what extent and in what ways this has been grassroots driven, by audience needs, or rather top down, by commercial interests?

## Critically reflecting on an experience society

For Pine and Gilmore (2011), the proliferation of staged experiences is the only way to overcome the limitations of commodity and service capitalism and create new added value for consumers. They argue that whereas ‘commodities are fungible, goods tangible, and services intangible, experiences are memorable’ (Pine and Gilmore, 2011: 17). This is because even though experiences end, their value lingers ‘in the memory of any individual who was engaged by the event’ (Pine and Gilmore, 2011: 18). Similarly, in audience and fan studies, we have seen writers, such as Jenkins, celebrating the rise of what he sees as a more participatory culture.

Jenkins (2006) suggests the development of a participatory culture is the result of three interrelated processes. First, Jenkins (2006: 136) suggests that there has been a rise in what he refers to as ‘transmedia’ texts – where narratives increasingly crosscut different media texts and platforms. This, then requires more active participation, as audiences need to follow multiple sources of content and narratives. For example, for football fans, this may include watching games on television, following and posting on various social media streams, reading reports and news on official and unofficial club websites, listening to podcasts, playing football-themed video games, and much more beyond.

Second, is the rise of digital media technologies that allow new forms of both top-down and bottom-up media engagement. For example, social media and on-demand television, have allowed media producers to distribute a much wider range of material, and have this made available for a longer period of time than was previously possible – such as football clubs showing ‘classic’ games on their television or social media channels. This then allows audiences to seek out a wider range of things to interest them, and also, find others who share their interests. However, and crucially, this is no longer (if it ever was) a one-way top-down process, but these technologies also allow audiences to actively engage with others and the texts they consume.

This, Jenkins (2006) links to a third development, which he terms the rise of the ‘DIY culture’. Jenkins sees this as something that began in specific subcultures, such as bloggers and video game modders (and we would add to this football fanzine creators and contributors), but this is now something that has spread from the margins to the mainstream. To produce media content is no longer subcultural or even unusual, but it has become an everyday and rather mundane activity, such as posting on social media.

Hence, for Jenkins, the rise of participatory culture has been driven by audience needs and technological advances, as much as (and probably more so) than media conglomerates’ desires to sell more content, services and (increasingly) experiences. There is, however, an alternative reading, and one that sees participatory culture and the rise of an experience society as part of the growing power and influence of neoliberalism.

McGuigan (2010: 117) suggests that neoliberalism ‘is a truly hegemonic phenomenon of our time’. At its core, neoliberalism is a *laissez-faire* economic and political system that encourages an open and free competitive market. There is nothing particularly new about neoliberalism, as it is an idea first developed in the early 20th century. However, it is after the economic crises of the 1970s that neoliberalism rose to prominence – replacing a more social-democratic model that had dominated much of mid-20th century. In this deregulated economy, it is the power of the consumer to choose that dominates – to choose where they will go, what they will do and who they will be. It is therefore a society and economy based upon participation. However, this is a participation that is required – as to not be an active participant in a neoliberal society, is to be, as Bauman (1998) highlights, a flawed consumer. Moreover, critiques of neoliberalism argue that this is not a participation that empowers, but rather individual choice leads to an increasingly individualised society, where recognition of our collective position and collective struggle has been lost in the pursuit of individual happiness (McGuigan, 2010). This is what Taylor (1995) feared. In a neoliberal football free market, supporters are able to choose any team from around the world to support, or possibly more accurately, football clubs, leagues, and media providers, are able to market their products and services more widely – such as Fleischmann and Fleischmann (2019) argued that many European clubs and leagues are increasingly looking towards more distant supporters in ‘emerging football markets’, such as China and other Asian nations, as new and more lucrative audiences. However, Taylor argued that this freedom to choose does not empower fans, but rather individualises and undermines any collective power they may have once had.

New digital technologies considerably extend the control and power of neoliberalism by increasing its market reach, and our and others’ (including new and old corporations’) capacities to track and monitor behaviour − greatly increasing self and social surveillance (Elis and Gill, 2018). Moreover, though it could be argued, as Jenkins does, that new digital media allow more opportunities for ‘grassroots’ content creation, Rivers and Ross (2019) suggested, as fan-created media channels become more successful, such as in the case of Arsenal Fan TV, they have a tendency to start to resemble the ‘corporate machine’ its creators originally set out to offer as an alternative.

Such arguments, therefore, add weight to the perspective that new digital media, rather than challenging, may be aiding, or even driving, a reconfiguration of capitalist modes of production and consumption. This shift then becomes even more pertinent and accelerated during a global pandemic and enforced isolation, where sport fans are primarily connecting with sport, and each other, through new digital technologies such as mobile devices. For, though engaging with others online, such as via social media, may give the impression of being in control and communal, ironically the more we seek connection and social interaction with others online, the more we disconnect from the material world and those around us – a condition that Turkle (2011) referred to as being ‘alone together’.

## Conclusion

With the return of live football towards the end of 2020, albeit initially without spectators in the stadia, *The Guardian* (2020b) posed the question ‘sport fans, what have you missed the most (or not at all) during lockdown?’ The article continued:

What do you miss most about the old normal? Gathering in the pub for a big Champions League night? That quickening walk to the ground as you hear the team being read over the Tannoy? Sitting down to watch Match of the Day with your family? Being part of an ongoing soap opera that punctuates your weekends, connects you to your friends and gives you something to talk about with people you have never met before? (*The Guardian, 2020b*: online).

Most of the activities listed in the quote above are communal, but to what extent was this already a fading idea of what sport fans do? Certainly, evidence would suggest that we are starting to see a decline in the viewing of sport with others via broadcast television and in contrast an increase in the consumption of sport alone via mobile devices. The question is, therefore, how may the COVID-19 pandemic have accelerated this? However, also, whether football clubs need fans in the stadium at all? As Ronay (2020) argues in *The Guardian* ‘it turns out that real life fans, while important, just aren't central enough to football's business model. Not at the top end anyhow’. Further evidence of this was provided in mid-2021 by the failed attempt of 12 of Europe's top football clubs to found a new break-away European Super league. Here, the BBC Sport reporter Dan Roan claimed that this was – at least in part – a result of the COVID-19 pandemic and the clubs’ desire to seek out new ‘fans of the future’ and rely less on what they saw as their ‘legacy fans’ – a term used to describe football's traditional, stadium-attending, supporters (Williams, 2021). As Ronay wrote in early April 2021, ‘football's biggest clubs have discovered what they already knew – that they don't actually need those troublesome, noisy humans in the stands, that this crisis is also an opportunity’ (Ronay, 2021). However, the fan protests that followed, and the subsequent backing down of the breakaway clubs and the end (or pausing) of their Super League dreams, may suggest that these clubs do still, at least for now, need their traditional match-attending supporters – but the question is, for how long? Many football clubs, in particular, the wealthier ones, are clearly looking towards their ‘fans of the future’, who will be more likely to connect with the sport via digital devices, which of course, enables them to consume the sport at distance, and allows football clubs to more readily tap into distant and lucrative ‘emerging football markets’, such as most notably, those in Asia (Fleischmann and Fleischmann, 2019).

Hence, for a new generation of sport fans, we will probably see a continued (and, we would suggest, now accelerated) shift towards the consumption of content via digital devices, as this offers the kind of individualised and personalised experiences that they have become accustomed to, but more importantly, this offers clubs, leagues and media providers new ways of connecting with new (and often more distant) audiences. Baudrillard (1993) may have famously predicted a future where football was played in empty stadia, but where he was wrong was to suggest that the consumption of this sport would shift solely to the mass spectacle of broadcast television. Instead, when the stadia did empty, it was to their individual digital and mobile devices that many supporters turned (or were directed) as a way to remain connected to the game that they love – and the global pandemic way well have accelerated this, already in process, shift in the consumption of football increasingly towards themed digital participatory experiences.
